# Clinical insights into *Candidatus* Mycoplasma haemominutum-associated anaemia in naturally infected cats: flea-associated risk and paradoxical clinicopathological findings

**DOI:** 10.1186/s13620-026-00359-x

**Published:** 2026-09-16

**Authors:** Mahmoud S. Safwat, Mohamed Abdel-Haleem, Omnia H. Refaei, Samah El-Sayed M., Ghada M. Khalil, M. E. Ali, El Shymaa A. Mohamed, Ahmed Orabi, Asmaa A. Rayan, Reham Karam, Mary A. N. Sargious, Mohamed I. Abdallah, Samah Eid, Mona M. Osman, Abdelhalim A. Elshawadfy, Ahmad Zaki Anwer

**Affiliations:** 1 Department of Internal Medicine and Infectious Diseases (Infectious Diseases), Faculty of Veterinary Medicine, Cairo University, Giza, 12211 Egypt; 2 Department of Botany and Microbiology, Faculty of Science, Zagazig University, Zagazig, 44519 Egypt; 3 Department of Clinical Pathology, Faculty of Veterinary Medicine, Cairo University, Giza, 12211 Egypt; 4 Department of Internal Medicine and Infectious Diseases (Internal Medicine), Faculty of Veterinary Medicine, Cairo University, Giza, 12211 Egypt; 5 Educational Veterinary Hospital, Zagazig University, Faculty of Veterinary Medicine, Zagazig, 44511 Egypt; 6 Department of Microbiology and Immunology, Faculty of Veterinary Medicine, Cairo University, Giza, 12211 Egypt; 7 Department of Microbiology and Immunology, Faculty of Veterinary Medicine, Assuit University, Assuit, 71526 Egypt; 8 Department of Virology, Faculty of Veterinary Medicine, Mansoura University, Mansoura, 35511 Egypt; 9 WEQAA Central Laboratory, National Centre for the Prevention and Control of Plant and Animal Diseases (WEQAA), Riyadh, 11454 Saudi Arabia; 10 Genome Research Unit (GRU), Animal Health Research Institute (AHRI), Agriculture Research Center (ARC), Dokki, Giza 12618 Egypt; 11 Department of Bacteriology, Animal Health Research Institute (AHRI), Agriculture Research Center (ARC), Dokki, Giza 12618 Egypt; 12 Mycoplasma Department, Animal Health Research Institute (AHRI), Agriculture Research Center (ARC), Dokki, Giza 12618 Egypt; 13 Department of Bacteriology, Mansoura Provincial Lab (AHRI-Mansoura), Animal Health Research Institute, Agriculture Research Center (ARC), Mansoura, Egypt

**Keywords:** Candidatus Mycoplasma haemominutum, Feline haemoplasma, Genetic characterisation, Pathophysiology, Pathogenesis, Phylogenetic analysis, Vector-borne transmission, Flea, Retroviruses, Egypt

## Abstract

**Background:**

The specific pathophysiological drivers and host–pathogen–environment factors that dictate the pathogenicity of *Candidatus* Mycoplasma haemominutum (CMhm) remain poorly defined, hindering accurate clinical interpretation and effective disease control. This study provides detailed mechanistic insights into CMhm-associated anaemia in naturally infected cats by integrating analyses of concurrent diseases and comprehensive clinicopathological panels, alongside molecular characterisation in an under-represented region.

Two groups of CMhm-infected cats (anaemic, *n* = 13; non-anaemic, *n* = 25) were systematically screened for 14 concurrent infectious, metabolic, or endocrine conditions, alongside extensive haematological, biochemical, and iron profile assessments. Multivariable logistic regression identified independent risk factors, while CMhm molecular diversity was assessed using 16S rRNA gene sequencing.

**Results:**

Multivariable analysis identified flea infestation as the sole significant driver of anaemia (OR 7.77; *P* = 0.04), underscoring a key vector-trigger role and contributing evidence to the ongoing debate on CMhm’s flea-borne transmission. Although specific conditions such as feline immunodeficiency virus, feline leukaemia virus, Mycoplasma haemofelis, Candidatus Mycoplasma turicensis, and hyperglycaemia were not statistically significant, they may still be biologically relevant. Notably, most cats in the non-anaemic group also had comorbidities, suggesting that disease stage or severity may be more critical than mere presence. Supporting this, the anaemic group showed significantly lower lymphocyte counts and a downward trend in other leucocytes. Anaemia appeared deceptively normocytic-normochromic and non-regenerative despite ongoing CMhm-induced haemolysis, indicating additional counteracting mechanisms. Detailed clinicopathological and concurrent disease investigations pointed to true iron deficiency (flea-related) and functional iron deficiency (inflammatory comorbidities) as contributors. The coexistence of these three opposing pathophysiological processes shaped the paradoxical haematological and iron profile. Molecular analysis identified three CMhm nucleotide sequence types (nSTs) new to the region.

**Conclusions:**

The results of this study suggest that CMhm-associated anaemia is likely a secondary condition, in which environmental triggers and host immune status shape disease manifestation. This study elucidates the complex pathophysiological mechanisms underlying anaemia, providing insights to enhance clinical diagnosis and inform control strategies.

## Background

Feline haemotropic *Mycoplasma* (FHM) species are blood-borne pathogens that infect domestic and wild felids worldwide [1]. They are small, gram-negative, cell-wall–devoid bacteria that adhere to the surface of red blood cells, causing a spectrum of clinical outcomes ranging from subclinical infection to severe life-threatening haemolytic anaemia [1]. Three major species are commonly found in cat populations globally: *Mycoplasma haemofelis* (Mhf), *Candidatus Mycoplasma haemominutum* (CMhm), and *Candidatus Mycoplasma turicensis* (CMt) [2]. Identification of FHM species in clinical samples is typically achieved using genus-specific 16 S rRNA PCR, followed by sequencing or species-specific PCR assays [2].

The exact transmission route of FHM remains uncertain; flea-borne transmission has been suggested, based on the clustering of infected cats in warmer areas or seasons [3–7] and the high prevalence of FHM DNA detected in fleas [8–13]. Cat-to-cat fighting is considered an alternative transmission route, given its high prevalence rate in males or those with an outdoor access lifestyle or those presenting with biting wounds [3, 5, 6, 14–19]. Following infection, the disease typically progresses through an acute phase, in which anaemia may develop, and then a chronic carrier state, characterised by low-level bacteraemia without clinical signs [2, 20–22]. Reactivation from the carrier state appears to be uncommon [20, 22], and the factors that trigger such reactivation remain poorly understood [23].

Despite CMhm being the most prevalent FHM species in cats worldwide [3, 5–7, 12–19], several important knowledge gaps and conflicting findings remain regarding CMhm-associated anaemia. These include CMhm’s role in anaemia (primary versus secondary), the influence of concurrent diseases on the development of anaemia, and the haematological and biochemical features of CMhm-associated anaemia. As a result, its true clinical presentation, interpretation, and implications for medical management remain largely undefined.

CMhm is generally regarded as a secondary pathogen with limited clinical significance [2]. Most experimental infections reported no anaemia [21, 24, 25]; moreover, larger epidemiological studies failed to find an association between CMhm infection and anaemia [5, 7, 14, 17–19, 26]. However, some experimental infection studies showed mild to moderate anaemia in a subset of animals [20, 22]. Similarly, a few naturally infected cats developed mild to severe anaemia without detectable concurrent diseases [23, 27, 28]. This inconsistency leaves the question of whether CMhm can ever act as a primary pathogen unresolved.

The contribution of concurrent diseases to CMhm-associated anaemia remains poorly understood. Epidemiological studies typically compared CMhm-infected versus non-infected cats, but this design cannot distinguish whether concurrent conditions facilitate anaemia or merely predispose cats to CMhm infection. Only limited experimental evidence has examined interactions with other pathogens, e.g., Mhf, feline leukaemia virus (FeLV), feline immunodeficiency virus (FIV) [20, 22, 24], and the influence of concurrent diseases in naturally infected populations is not well defined.

Understanding the characteristics and mechanisms of CMhm-associated anaemia remains challenging. Experimental infections in pathogen-free cats have shown that CMhm alone can induce anaemia, typically regenerative and macrocytic [20, 22]; however, these studies do not reflect real-world conditions where concurrent infections and host immune diversity may shape the anaemia differently. Epidemiological comparisons of CMhm-infected versus non-infected cats are also limited, as the “infected” groups often include a majority of healthy carriers, diluting the signal of anaemia and obscuring the true haematological profile of affected cats. Furthermore, case reports and small case series [23, 27, 28], while informative, are largely descriptive and lack the statistical power needed to characterise the nature of CMhm-associated anaemia.

Just as CMhm’s clinical significance is uncertain, its molecular diversity is poorly defined in several parts of the world. In Egypt, for example, only two partial 16 S rRNA gene sequences have been published, limiting the global phylogenetic understanding [29].

The present study addresses these gaps by examining a cohort of CMhm-infected cats for concurrent infectious, metabolic, and endocrine disturbances and by detailing comprehensive clinical-pathology profiles, comparing the obtained results between anaemic and non-anaemic cats within the cohort. In addition, it provides the largest set of partial 16 S rRNA gene sequences from Egypt to date. This combined approach helps to (a) assess whether CMhm may act as a primary pathogen in the absence of other diseases; (b) identify which concurrent diseases are most frequently associated with anaemia, consistent with a secondary-pathogen role; (c) characterise the real-world haematological and biochemical profile of CMhm-associated anaemia at both population and individual levels; and (d) expand local molecular data to strengthen the global phylogenetic framework.

## Materials and methods

### Study population and sampling

A total of 38 CMhm naturally infected cats were included in the current study. These cats represented all CMhm-infected cats identified in our recent survey on haemotropic *Mycoplasma* spp. in Egyptian cats, randomly selected from three governorates between December 2022 and April 2024 for research purposes rather than routine diagnostic care [14]. Some cats were infected only with CMhm, while others were coinfected with other *Mycoplasma* spp. including Mhf and CMt. CMhm infection had been confirmed in these cats using genus- and species-specific conventional PCR assays, as summarised in Table 1.

**Table 1:** PCR assays used for the detection of CMhm and other pathogens in cats, with primer sequences, expected amplicon sizes, annealing temperature (*T*_a_), references, and positive controls

Pathogen^a^	Target gene^b^	Specificity	Primer sequence ^c^	Size (bp) ^d^	*T*_a_ (°C)	Reference	Positive controls (previously characterised sequences, vaccines, or synthetic gBlock)
Pathogens for which the study population was tested in previous or ongoing surveys							
*Mycoplasma* spp.	*16 S rRNA*	Genus-specific	F. ACGAAAGTCTGATGGAGCAATA	170**/** 193	60	[30]	GenBank acc. no. PQ328756 and PQ328758 [14]
			R. ACGCCCAATAAATCCGGATAAT				
Mhf	*16 S rRNA*	Species-specific	F. ATGCCCCTCTGTGGGGGATAGCCG	275	58	[31]	Acc. nos. PQ328756 [14]
			R. ATGGTATTGCTCCATCAGACTTTCG				
CMhm	*16 S rRNA*	Species-specific	F. CTGGGAAACTAGAGCTTCGCGAGC	202	58	[31]	Acc. no. PQ328758 [14]
			R. ATGGTATTGCTCCATCAGACTTTCG				
CMt	*16 S rRNA*	Species-specific	F. GTATCCTCCATCAGACAGAA	488	55	[32]	Acc. nos. PQ328759 [14]
			R. CGCTCCATATTTAATTCCAA				
FIV	*env3-5*	Validated for subtypes A and B	F. GTACAGACCCATTACAAATC	776	53	[33]	Acc. no. OR882079 [34]
			R. CTGCCACTGGGTTATACCAA				
FeLV	*LTR*	Any subgroup	F. AACAGCAGAAGTTTCAAGGCC R. TTATAGCAGAAAGCGCGCG	135	60	[35]	Nobivac FeLV inactivated vaccine (Rickard strain; MSD, Netherlands)
Pathogens for which the study population was tested for the first time in this study							
PPVC1	*VP2*	FPV, CPV-2	F. CCATGGAAACCAACCATACC	717	55	[36]	Acc. nos. PP663049 and MT636872 [37, 38]
			R. AGTTAATTCCTGTTTTACCTCCAA				
Piroplasms	*18 S rRNA*	Any (*Cytauxzoon*, *Babesia*, etc.)	F. GACACAGGGAGGTAGTGACAAG	415–447	52	[39]	Acc. no. MW557303 [40]
			R. CTAAGAATTTCACCTCTGACAGT				
*Anaplasma* spp.	*groEL*	Genus-specific	F. TGGTATGCAGTTTGATCGC	625	60	[41]	Acc. no. MW556745 [40]
			R. TCTACTCTGTCTTTGCGTTC				
*Bartonella* spp.	*ssrA*	Genus-specific	F. GCTATGGTAATAAATGGACAATGAAATAA	301	60	[42]	Synthetic gBlock (IDT, USA)
			R. GCTTCTGTTGCCAGGTG				
*Ehrlichia* spp.	*16 S rRNA*	Genus-specific	F. GAGGATTTTATCTTTGTATTGTAGCTAAC	210	58	[43]	Synthetic gBlock (IDT, USA)
			R. TGTAAGGTCCAGCCGAACTGACT				
CMhm genetic characterisation and phylogenetic analysis							
*Mycoplasma* spp.	*16 S rRNA*	Genus-specific	F. ATACGGCCCATATTCCTACG	595**/** 618	60	[44]	Acc. no. PQ328756 and PQ328758 [14]
			R. TGCTCCACCACTTGTTCA				

^a^ Pathogen abbreviations: *CMhm Candidatus* Mycoplasma haemominutum, *CMt Candidatus* Mycoplasma turicensis, *FeLV* feline leukaemia virus, *FIV* feline immunodeficiency virus, *Mhf Mycoplasma haemofelis, PPVC1 protoparvovirus carnivoran* 1

^b^ Target gene/region definitions (*16 S rRNA*: Small-subunit ribosomal RNA gene of prokaryotes (bacteria); *env*3-5 gene encoding the highly variable regions 3–5 of the envelope gene; *LTR* long terminal repeat regions, *VP2* the major capsid protein, *18 S rRNA,* Small-subunit ribosomal RNA gene of eukaryotes (protozoa), *groEL* Gene encoding the bacterial chaperonin GroEL (heat shock protein), *ssrA* Gene encoding transfer-messenger RNA)

^c^ Notes on PCR types: all conventional except FeLV: real-time PCR with probe FAM-CCAGCAGTCTCCAGGCTCCCCA-TAMRA

^d^ Notes on amplicon sizes: Three PCR assays can yield variable amplicon sizes depending on the species amplified: the *Mycoplasma* spp. PCR (Jensen et al. [30]) yields 170 bp for Mhf/CMt and 193 bp for CMhm; the Piroplasms PCR assay (Liu et al. [39]) gives a wide range of amplicon sizes from 415 bp (*Babesia vogeli*, *Babesia canis*, *Babesia rossi*), 440 bp (*Cytauxzoon* spp.), 447 bp (*Babesia felis*, *Babesia leo*, *Babesia lengau*); the *Mycoplasma* spp. PCR assay (Criado-Fornelio et al. [44]) produces 595 bp for Mhf/CMt and 618 bp for CMhm

For the current investigation, cats were classified into two groups based on anaemia status: anaemic (HCT < 30%; *n* = 13) and non-anaemic (*n* = 25). In the anaemic group, the majority of cats were shelter-housed (*n* = 11), with 2 client-owned; their ages were either ≤ 6 years (*n* = 6) or > 6 years (*n* = 7). This group included 12 males (7 intact, 5 castrated) and 1 intact female. Similarly, in the non-anaemic cohort, most cats were shelter-housed (*n* = 22), with only 3 client-owned cats; their ages were either ≤ 6 years (*n* = 10) or > 6 years (*n* = 15). This group comprised 23 males (14 castrated, 9 intact) and 2 intact females.

Complete blood count (CBC) analyses were performed within 6 h of blood collection using fresh whole blood samples during the original survey. During that same original survey, FIV and FeLV testing was conducted simultaneously on fresh whole blood samples. In the present study, archived serum and DNA extracts, stored at − 20 °C for 6–24 months, were used for biochemical analyses and molecular screening of additional pathogens, respectively.

Ethical approval for this study was obtained from the Institutional Animal Care and Use Committee of the Faculty of Veterinary Medicine, Cairo University (Vet CU110520251076). Oral consent was obtained from owners and shelter directors during the original survey.

### Haematological investigations

Complete blood count (CBC) results of all 38 CMhm-infected cats are reported in detail in this study (see Results section; Table 3). These data were previously incorporated into the mean or median values of various CBC parameters in broader groups in the original survey [14]. The CBC included red blood cell (RBC) count, haemoglobin (Hgb) concentration, haematocrit (HCT), mean corpuscular volume (MCV), mean corpuscular haemoglobin concentration (MCHC), and total leucocyte count (TLC), which were measured using the ABC™ Animal Blood Counter (ABC Vet, France). Differential leucocyte counts were assessed manually by microscopic examination of Diff-Quik-stained peripheral blood smears, and platelet counts were determined using the direct manual dilution method with a haemocytometer. Reticulocyte aggregates were identified and counted microscopically on blood smears prepared from whole blood mixed with the supravital stain new methylene blue. Anaemia severity was classified by HCT (%) as mild (21–29%), moderate (15–20%), or severe (< 15%). Anaemia was further characterised using RBC indices: MCV > 55 fL (macrocytic), 39–55 fL (normocytic), and < 39 fL (microcytic); MCHC > 36 g/dL (hyperchromic), 30–36 g/dL (normochromic), and < 30 g/dL (hypochromic). Regeneration was assessed by absolute reticulocyte aggregate count, with ≥ 0.06 × 10⁶/µL considered regenerative and counts < 0.06 × 10⁶/µL non-regenerative.

### Biochemical analyses

Serum concentrations of creatinine, fructosamine, total thyroxine (T4), total bilirubin (TBIL), alanine aminotransferase (ALT), total proteins (TP), and albumin (ALB) were measured in all 38 CMhm-infected cats. Creatinine and fructosamine were measured using IDEXX slides analysed by the Catalyst One Chemistry Analyser(IDEXX, USA). T4 was measured using Vcheck T4 kits analysed by the Vcheck V200 Analyser (BioNote, South Korea). TBIL, ALT, TP, and ALB were measured using a semi-automated biochemical analyser (Robonik, India) with specific kits (Spectrum Diagnostics, Egypt). Globulin (GLOB) concentrations were calculated by subtracting albumin from total proteins.

Serum iron studies, including serum iron, ferritin, and total iron-binding capacity (TIBC), were performed only in the anaemic group because sufficient residual serum volume was not available for most non-anaemic cats. Ferritin was measured using an immunoassay kit (Biomed, Egypt), whereas serum iron and TIBC were measured using colourimetric kits (serum iron: Spectrum Diagnostics, Egypt; TIBC: Biomed, Egypt), and all readings were obtained with a semi-automated photometer (Photometer 4040 V5, Germany). Transferrin saturation (TSAT) was calculated using the standard equation: .

$$TSAT\:\left(\%\right)=\:\frac{Serum\:iron\:(\upmu{g}/dL)}{TIBC\:(\upmu{g}/dL)}\:X\:100$$

### Investigation of other pathogens

All CMhm-infected cats were examined for concurrent infection with other pathogens, including other haemotropic *Mycoplasma* spp. (Mhf and CMt), fleas, upper respiratory infection (URI), FIV, FeLV, *protoparvovirus carnivoran* 1 (PPVC1), Piroplasms (*Cytauxzoon* spp. and *Babesia* spp.), *Anaplasma* species, *Bartonella* species, and *Ehrlichia* species.

Mhf and CMt were detected using genus- and species-specific conventional PCR assays during the original survey [14]. Fleas were assessed by systematic combing of the cats’ coats, and feline URI was diagnosed based on clinical signs (nasal discharge, sneezing, conjunctivitis, oral ulceration). Data on fleas and URI were collected during the original survey but have not been previously reported.

FIV detection was performed as part of another recent survey, where infection status was determined based on a combination of two antibody-based point-of-care (PoC) kits (SNAP^®^ FIV Ab/FeLV Ag kit [IDEXX, USA] and Anigen^®^ FIV Ab/FeLV Ag kit [BioNote, South Korea]) and a conventional PCR assay. Cats were considered FIV-infected when at least two diagnostic techniques agreed or when PCR results were confirmed by sequencing. Subtyping was successful in only 10 FIV-infected cats in the original survey [34], all of which harboured the novel subtype “X-EGY.” Five of these were among the cats concurrently infected with both CMhm and FIV that are included in the present study.

Similarly, FeLV infection status was established during an ongoing large-scale survey in our laboratory using the same two PoC kits (detecting FeLV p27 antigen) and a real-time PCR assay. Cats were deemed FeLV-infected when a consensus of at least two diagnostic methods was obtained, or when PCR positivity was confirmed by sequencing.

The remaining pathogens (PPVC1, piroplasms, *Anaplasma* spp., *Bartonella* spp., and *Ehrlichia* spp.) were investigated for the first time in this study using conventional PCR assays. Cats testing positive by PCR were considered infected, and sequencing was intended for species-level identification of any positive samples. All PCR methodologies used to investigate concurrent pathogens are given in Table 1.

### Molecular characterisation and phylogenetic analysis of CMhm

In our previous survey [14], both genus- and CMhm-specific PCR assays produced short amplicons. In the present study, a PCR assay targeting a longer fragment was applied to all 38 CMhm-infected cats to enable molecular characterisation and phylogenetic analysis (see Table 1). Only 10 samples yielding clear, strong bands on agarose gel were selected for sequencing. Bands were purified using the QIAquick^®^ PCR Purification Kit (Qiagen, Germany) following the manufacturer’s instructions. Purified products were directly sequenced on both strands using the PCR primers and the BigDye Direct Cycle Sequencing Kit (ThermoFisher, USA) according to the manufacturer’s protocols.

**Fig. 1:**
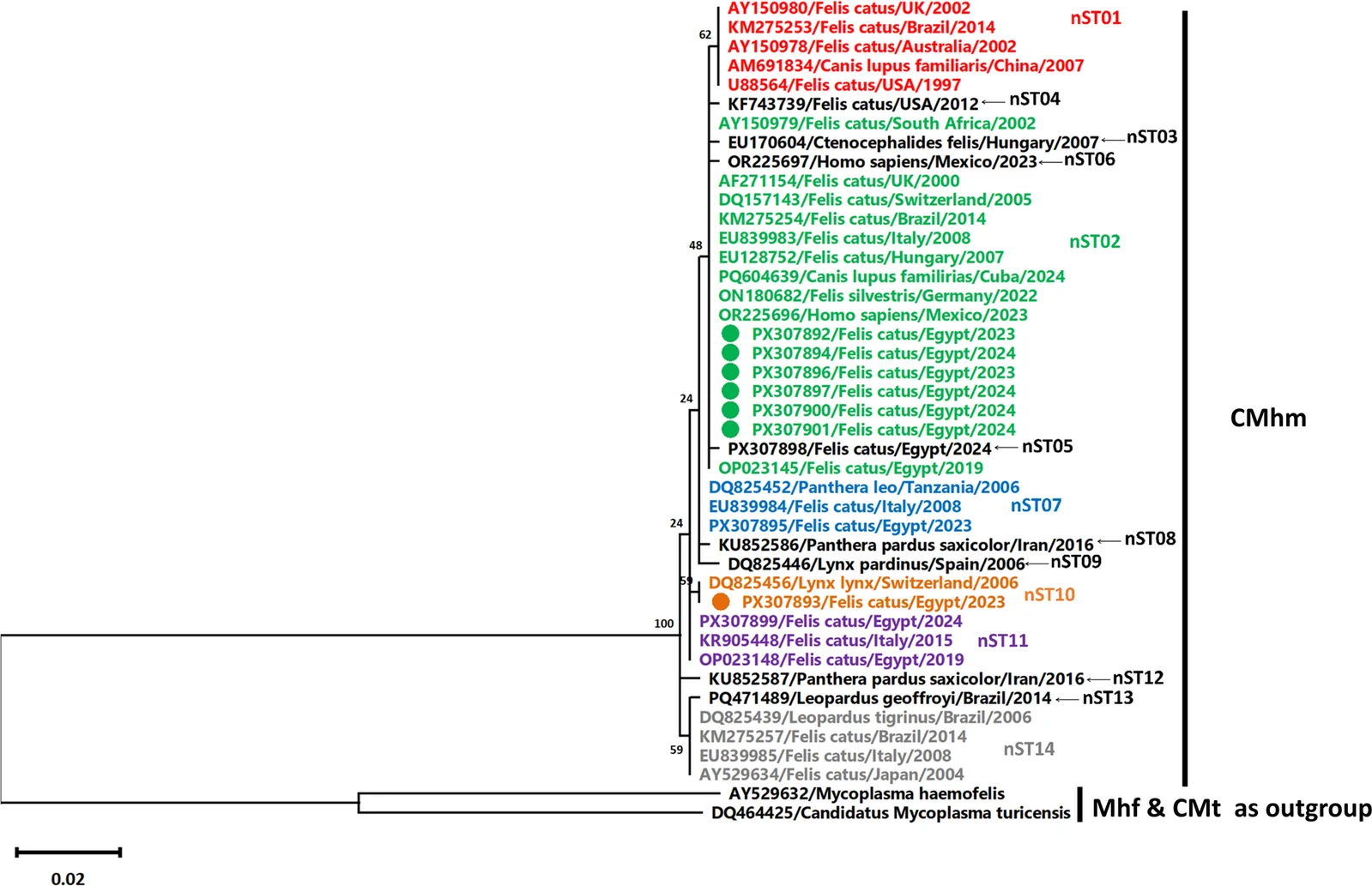
Maximum-likelihood phylogenetic tree of *Candidatus* Mycoplasma haemominutum partial *16 S rRNA* gene sequences (550 nucleotides). The tree is rooted with *Mycoplasma haemofelis* (Mhf) and *Candidatus* Mycoplasma turicensis (CMt). The Tamura 3-parameter model with Gamma distribution was used, and the tree was constructed with 1,000 bootstrap replicates. The dataset includes 10 Egyptian sequences generated in this study (labelled with circles) and 31 reference sequences. For each sequence, the GenBank accession number, host species, country, and submission or collection date are indicated directly on the tree. The 14 nucleotide sequence types (nSTs) identified in this study are also shown. The scale bar represents the number of nucleotide substitutions per site

The sequences generated in this study (*n* = 10) were edited and assembled using BioEdit software version 7.2.5 and deposited in GenBank under accession numbers PX307892–PX307901. These sequences were analysed together with 31 reference CMhm sequences retrieved from GenBank (accessed in August 2025), resulting in a combined dataset of 41 sequences used for nucleotide sequence type (nST) assignment and phylogenetic analysis. Reference sequences were selected to represent diverse countries, collection times, and host species. Detailed information on reference sequences, including accession numbers, country, host, and collection or submission date, is provided in the phylogenetic tree (see Results, Fig. 1).

All 41 sequences in the combined dataset were assigned to different nucleotide sequence types, with each nST containing sequences that were identical. Pairwise mutation and nucleotide identity matrices were generated for these nSTs using BioEdit. The evolutionary history of the 41-sequence dataset was inferred from a maximum likelihood (ML) tree constructed using the Tamura 3-parameter model with gamma distribution (T92 + G) in MEGA version 11. The T92 + G model was identified as the best-fit model for our dataset using MEGA’s “Find Best DNA/Protein Model” tool. Tree branch reliability was assessed by bootstrap analysis with 1000 iterations. The tree was rooted using two outgroup sequences (Mhf, AY529632 and CMt, DQ464425) and subsequently edited in FigTree version 1.4.4.

### Statistical analysis

All statistical analyses were performed using SPSS software. CMhm-infected anaemic and non-anaemic groups were compared for statistically significant differences in categorical variables, including demographic characteristics and concurrent infectious or metabolic/endocrine conditions. Demographic factors included age (≤ 6 years vs. >6 years), sex (male vs. female), neuter status of males (intact vs. castrated), and housing (shelter-housed vs. client-owned). Concurrent infectious diseases assessed were the presence versus absence of fleas, URI, Mhf, CMt, FIV, FeLV, PPVC1, piroplasms, *Anaplasma* spp., *Bartonella* spp., and *Ehrlichia* spp. Metabolic and endocrine disturbances included azotemia (elevated vs. within-reference-interval creatinine concentrations), hyperglycaemia (elevated vs. within-reference-interval fructosamine concentrations), and hyperthyroidism (elevated vs. within-reference-interval T4 concentrations). Comparisons were performed using the Chi-square test for cell frequencies > 5 or Fisher’s exact test for cell frequencies < 5. Variables with *P* < 0.25 in univariable analyses were considered candidates for multivariable analysis. Possible collinearity between independent variables was assessed using Pearson’s correlation, and variables with correlation coefficients > 0.9 were excluded. Multivariable analysis was performed using logistic regression (enter method). Variables with *P* < 0.05 were retained in the final model, and their odds ratios (ORs) with 95% confidence intervals (CIs) were reported.

Continuous haematological and serum biochemical variables were analysed as follows. Normality of distribution was assessed separately within each group (anaemic and non-anaemic) using the Shapiro-Wilk test. Also, outliers were identified within each group using Tukey’s fences (*r* = 1.5). Variables with a normal distribution and no outliers were summarised as mean ± standard deviation, while variables that were non-normally distributed or contained outliers were summarised as median (Q1, Q3). Equality of variances between the two groups was evaluated using Levene’s test. Differences in means between groups were assessed using the Student’s t-test when variances were equal or the Welch’s t-test when variances were unequal. Differences in medians were assessed using the Mann-Whitney U test. Variables with *P* < 0.05 were considered statistically significant.

## Results

### Determinants of CMhm-induced anaemia

All CMhm-infected anaemic cats (13/13, 100%) and most non-anaemic cats (22/25, 88%) had at least one concurrent disease (*P* = 0.53). Table 2 summarises the findings from the univariate and multivariable analyses investigating determinants of CMhm-associated anaemia. Demographic factors (age, sex, neuter status, housing) were not associated with CMhm-induced anaemia. Among infectious and metabolic/endocrine factors, fleas, FIV, Mhf, and hyperglycaemia met the criteria for inclusion in the multivariable model (*P* < 0.25; no collinearity detected). In the final model, only flea infestation was significantly associated with CMhm-induced anaemia (OR 7.77; 95% CI 1.15–56).

**Table 2:** Univariate and multivariate analyses of host factors, infectious diseases, and metabolic/endocrine states associated with CMhm-related anaemia

Variable	Categories	Anaemic group (13) *n* (%)	Non-anaemic group (25) *n* (%)	Univariate *P* value ^b^	Multivariate	
					*P* value ^c^	AOR (95% CI)
Host factors						
Age	> 6 years	7 (53.8)	15 (60.0)	0.7155	-	-
	≤ 6 years	6 (46.2)	10 (40.0)			
Sex	Males	12 (92.3)	23 (92.0)	1	-	-
	Females	1 (7.7)	2 (8.0)			
Neutering status (males)	Intact	7 (58.3)	9 (39.1)	0.337	-	-
	Castrated	5 (41.7)	14 (60.9)			
Housing	Shelter	11 (84.6)	22 (88.0)	1	-	-
	Private	2 (15.4)	3 (12.0)			
Infectious diseases						
FIV	Present	8 (61.5)	9 (36.0)	0.178	0.368	
	Absent	5 (38.5)	16 (64.0)			
FeLV	Present	1 (7.7)	0 (0.0)	0.3421	-	-
	Absent	12 (92.3)	25 (100.0)			
Mhf	Present	4 (30.8)	3 (12.0)	0.203	0.254	-
	Absent	9 (69.2)	22 (88.0)			
CMt	Present	3 (23.1)	3 (12.0)	0.392	-	-
	Absent	10 (76.9)	22 (88.0)			
Fleas	Present	5 (38.5)	2 (8.0)	0.034	0.043	7.77 (1.15 -56)
	Absent	8 (61.5)	23 (92.0)			
URI	Present	7 (53.8)	11 (44.0)	0.564	-	-
	Absent	6 (46.2)	14 (56.0)			
Metabolic/endocrine states ^a^						
Hyperglycaemia	Present	6 (46.2)	4 (16.0)	0.06	0.327	-
	Absent	7 (53.8)	21 (84.0)			
Azotaemia	Present	0 (0.0)	2 (8.0)	0.538	-	-
	Absent	13 (100.0)	23 (92.0)			

Hyperthyroidism and other pathogens (*protoparvovirus carnivoran*1, piroplasms, *Anaplasma* spp., *Bartonella* spp., and *Ehrlichia* spp.) were absent in both groups and are therefore not included in the analysis

*Abbreviations*: *AOR* adjusted odds ratio, *CI* confidence interval, *CMhmCandidatus* Mycoplasma haemominutum, *CMtCandidatus* Mycoplasma turicensis, *FeLV* feline leukaemia virus, *FIV* feline immunodeficiency virus, *MhfMycoplasma haemofelis,URI* upper respiratory infection

^a^ Metabolic/endocrine states were identified based on a single laboratory measurement above the reference range: azotaemia (creatinine, 0.8–2.4 mg/dL), hyperglycaemia (fructosamine, 191–349 µmol/L), and hyperthyroidism (T4, 0.8–4.7 µg/dL)

^b^ Variables with *P* < 0.25 in univariate analysis were considered as candidates for the multivariable model

^c^ Variables with *P* < 0.05 were retained in the final multivariable model

### Clinical and clinicopathological profile of CMhm-infected cats

The CMhm-infected anaemic group exhibited significantly lower mean HCT and median Hgb values than non-anaemic carriers, though these remained mildly below the reference interval (Table 3). RBC median values were also significantly lower in anaemic cats, albeit still at the lower end of the reference interval. RBC indices (MCV and MCHC) were within reference ranges and did not differ significantly between groups. Among leucocytes, only lymphocyte counts were significantly lower in anaemic cats, remaining within the reference interval, while TLC, neutrophils, and monocytes were lower but not statistically significant. Biochemically, anaemic cats showed elevated TBIL and fructosamine, whereas ALT and creatinine remained within the reference interval.

**Table 3:** Comparison of continuous haematological and serum biochemical variables between CMhm-infected anaemic and non-anaemic cats, with statistical significance

Parameter	Reference range	CMhm anaemic group Mean ± SD or Median (Q1, Q3)	CMhm-infected non-anaemic group Mean ± SD or Median (Q1, Q3)	*P* value ^a^
HCT (%)	30–45	22.10 ± 4.10	37.70 ± 5.10	**< 0.001**
Hgb (g/dL)	9.8–15.4	8.20 (4.45, 8.60)	12.71 (11.12, 13.72)	**< 0.001**
RBCs (10⁶/µL)	5–10	5.25 (4.08, 6.30)	9.60 (7.20, 11.68)	**< 0.001**
MCV (fL)	39–55	39.19 (35.46, 52.16)	42.31 (35.17, 49.86)	1.00
MCHC (g/dL)	30–36	31.65 (28.70, 36.50)	31.43 (28.27, 37.51)	0.95
TLC (10³/µL)	5–19.5	15.00 (11.50, 24.50)	18.60 (13.90, 26.10)	0.28
Neutrophils (10³/µL)	2.5–12.5	7.44 (3.78, 19.50)	11.34 (6.84, 16.35)	0.58
Lymphocytes (10³/µL)	1.5–7	3.17 (2.22, 5.33)	5.17 (3.73, 7.45)	**0.04**
Monocytes (10³/µL)	0–0.9	0.48 (0.06, 1.22)	0.87 (0.52, 1.96)	0.16
Eosinophils (10³/µL)	0–0.8	0.90 (0.19, 1.24)	0.79 (0.14, 2.35)	0.47
Platelets (10³/µL)	175–600	312.50 (172.50, 475.00)	309.00 (212.50, 415.00)	0.77
Total protein (g/dL)	5.7–8.9	9.50 (7.63, 10.43)	9.80 (8.80, 11.00)	0.74
Albumin (g/dL)	2.3–3.9	2.32 (2.15, 2.50)	2.35 (2.27, 2.52)	0.33
Globulin (g/dL)	2.8–5.1	7.47 (5.32, 7.74)	7.03 (6.19, 8.66)	0.80
ALT (U/L)	12–130	18.42 (10.06, 29.08)	24.03 (18.74, 33.81)	0.18
Total bilirubin (mg/dL)	0–0.5	0.58 (0.32, 1.76)	0.24 (0.16, 0.54)	**0.02**
Creatinine (mg/dL)	0.8–2.4	0.96 (0.76, 1.46)	1.17 (1.02, 1.41)	0.30
Fructosamine (µmol/L)	191–349	355.00 (209.00, 456.00)	210.00 (184.00, 290.00)	**0.04**
T4 (µg/dL)	0.8–4.7	1.50 (0.94, 1.72)	1.46 (1.23, 1.88)	1.00
Reticulocytes (10⁶/µL)^b^	< 0.06	0.05 (0.03, 0.16)	-	-
Iron (µg/dL)c	80–180	105.50 (73.00, 222.00)	-	-
Ferritin (µg/L)^c^	50–200	88.00 (14.00, 245.00)	-	-
TIBC (µg/dL)^c^	222–430	506.00 (395.00, 720.00)	-	-
TSAT (%)^c^	20–56	20.70 (12.40, 30.90)	-	-

*Abbreviations*: *ALT* alanine aminotransferase, *CMhmCandidatus* Mycoplasma haemominutum, *HCT* haematocrit, *Hgb* haemoglobin, *MCHC* mean corpuscular haemoglobin concentration, *MCV* mean corpuscular volume, *Q1 and Q3* quartile 1 and 3, respectively, *RBCs* red blood cells, *SD* standard deviation, *TIBC* total iron-binding capacity, *TLC* total leucocyte count, *TSAT* transferrin saturation, *T4* thyroxine

^a^ Variables with *P* values < 0.05 were considered statistically significant and are shown in bold

^b^ Reticulocyte counts were available for anaemic cats only

^c^ Iron profile was obtained for 10 of the 13 anemic cats; no iron data were available for non-anemic cats

All cell counts are presented as absolute numbers

Individual profiles of anaemic cats (Table 4) revealed mild to moderate anaemia, ranging from regenerative macrocytic to non-regenerative microcytic forms, and highlighted paradoxical clinical pathology patterns at the individual level.

**Table 4:** Clinical, haematological, and biochemical profiles of CMhm-infected anaemic cats

Catno.	Conc. Dx	Hemogram						Leukogram					PLT 10³/µL	TBIL mg/dL	Iron profile				Protein profile		
		HCT %	Hgb g/dL	RBCs 10⁶/ µL	MCV fL	MCHC g/dL	Retic 10⁶/ µL	TLC 10³/ µL	*N* 10³/ µL	L 10³/ µL	M 10³/ µL	E 10³/ µL			Iron µg/dL	Fer µg/L	TIBC µg/dL	TSAT %	TP g/dL	GLOB g/dL	ALB g/dL
Regenerative, macrocytic normochromic, mild anaemia																					
278	Mhf, CMt & URI	**26**↓	**9.27**↓	**3.95**↓	**65.8**↑	35.7	**0.15**↑	15.0	5.4	**7.50**↑	**1.20**↑	**0.90**↑	363	**1.48**↑	129	**245**↑	**518**↑	24.9	**9.5**↑	**7.2**↑	2.3
Regenerative, normocytic normochromic, mild-moderate anaemia																					
43	Mhf, CMt, FIV & fleas	**18**↓	**6.26**↓	**4.20**↓	42.9	34.8	**0.25**↑	15.4	12.3	2.46	0.31	0.31	**150**↓	**2.16**↑	158	76	395	40.0	**13.6**↑	**11.5**↑	**2.1**↓
217	URI	**26**↓	**8.20**↓	5.25	49.5	31.5	**0.18**↑	12.4	7.4	1.98	**1.24**↑	**1.74**↑	300	0.40	**30**↓	**12**↓	330	**9.1**↓	7.6	5.3	2.3
Regenerative, microcytic hyperchromic, mild anaemia																					
187	FIV	**23**↓	**8.59**↓	6.25	**36.8**↓	**37.3**↑	**0.18**↑	12.2	7.3	3.17	0.24	**1.46**↑	325	**0.55**↑	82	**34**↓	**495**↑	**16.6**↓	**10.6**↑	**8.3**↑	2.3
Non-regenerative, microcytic hyperchromic, mild-moderate anaemia																					
159	FIV & fleas	**20**↓	**8.60**↓	5.25	**38.1**↓	**43.0**↑	0.04	6.2	2.9	2.98	0.12	0.25	**2225**↑	**0.60**↑	**490**↑	**390**↑	**930**↑	52.6	**9.5**↑	**7.5**↑	**2.0**↓
251	FIV & URI	**23**↓	**8.60**↓	6.35	**36.2**↓	**37.4**↑	0.02	**22.0**↑	**18.5**↑	3.50	0.00	0.00	**150**↓	**1.06**↑	**77**↓	**14**↓	**620**↑	**12.4**↓	5.9	3.7	**2.2**↓
Non-regenerative, normocytic normochromic, mild anaemia																					
160	Mhf, CMt, FIV & URI	**26**↓	**8.23**↓	**4.85**↓	53.6	31.7	0.04	8.0	6.2	**1.12**↓	0.48	0.16	525	0.24	**222**↑	100	**720**↑	30.8	**10.5**↑	**7.8**↑	2.7
200	FIV & fleas	**29**↓	**9.60**↓	7.40	39.2	33.1	0.04	12.2	4.9	5.61	0.73	**0.98**↑	425	**3.00**↑	**254**↑	**299**↑	**820**↑	30.9	**10.0**↑	**7.4**↑	2.6
Non-regenerative, normocytic hypochromic, moderate anaemia																					
38	Fleas & URI	**18**↓	**4.63**↓	**3.55**↓	50.7	**25.7**↓	0.05	**35.6**↑	**27.1**↑	2.85	**4.30**↑	**1.39**↑	300	0.39	–	–	–	–	**10.4**↑	**7.7**↑	2.7
Non-regenerative, microcytic hypochromic, mild-moderate anaemia																					
14	FIV, fleas & septic peritonitis	**17**↓	**4.30**↓	**4.90**↓	**34.7**↓	**25.3**↓	0.05	**53.9**↑	**51.7**↑	**1.08**↓	0.00	**1.08**↑	300	0.20	**43**↓	119	380	**11.0**↓	7.6	5.3	2.3
108	Mhf, FIV, URI & stomatitis	**18**↓	**5.30**↓	5.85	**30.8**↓	**29.4**↓	0.02	**27.0**↑	**20.5**↑	4.32	**1.08**↑	**1.08**↑	**1200**↑	**2.04**↑	**73**↓	**9**↓	**468**↑	**15.6**↓	**9.5**↑	**7.5**↑	**2.0**↓
286	URI	**25**↓	**6.99**↓	14.20	**17.6**↓	**27.9**↓	0.04	18.0	10.8	5.04	**2.16**↑	0.00	**170**↓	0.22	–	–	–	–	7.4	5.1	2.3

*Abbreviations*: *ALB* albumin, *CMhmCandidatus* Mycoplasma haematominutum, *CMtCandidatus* Mycoplasma turicensis, *E* eosinophils, *Fer* ferritin, *FIV* feline immunodeficiency virus, *GLOB* globulin, *HCT* haematocrit, *Hgb* haemoglobin, *L* lymphocytes, *M* monocytes, *MCHC* mean corpuscular haemoglobin concentration, *MCV* mean corpuscular volume, *MhfMycoplasma haemofelis,N* neutrophils, *PLT* platelets, *RBCs* red blood corpuscles, *Retic* reticulocytes, *TBIL* total bilirubin, *TIBC* total iron binding capacity, *TLC* total leucocytic count, *TP* total protein, *TSAT* transferrin saturation, *URI* upper respiratory infection

Reference ranges: ALB, 2.3–3.9 g/dL; E, 0–0.8 × 10³/µL; Fer, 50–200 µg/L; GLOB, 2.8–5.1 g/dL; HCT, 30–45%; Hgb, 9.8–15.4 g/dL; Iron, 80–180 µg/dL; L, 1.5–7 × 10³/µL; M, 0–0.9 × 10³/µL; MCHC, 30–36 g/dL; MCV, 39–55 fL; N, 2.5–12.5 × 10³/µL; PLT, 175–600 × 10³/µL; RBCs, 5–10 × 10⁶/µL; Retic, < 0.06 × 10⁶/µL; TBIL, 0–0.5 mg/dL; TIBC, 222–430 µg/dL; TLC, 5–19.5 × 10³/µL; TP, 5.7–8.9 g/dL; TSAT, 20–56%

All cats had serum ALT and creatinine within the reference ranges (ALT: 12–130 U/L; Creatinine: 0.8–2.4 mg/dL)

↑ and ↓ symbols indicate values above or below the reference range, respectively; (–) indicates not tested

Clinically, the most consistent findings among anaemic cats were lethargy and emaciation. Fever was uncommon and recorded in only two cats, while overt icterus was not observed in any case despite the mild increase in TBIL concentrations. In addition to anaemia-associated manifestations, concurrent disease-related clinical signs were also common, particularly upper respiratory tract signs including mucopurulent nasal or ocular discharge, sneezing, and conjunctivitis, which were observed in seven cats. Stomatitis was identified in only one cat. The most severe clinical case involved a cat co-infected with CMhm and FeLV, monitored over one year for haematology and pathogen detection (Table 5). The cat exhibited persistent, non-regenerative macrocytic normochromic anaemia with progressive thrombocytopenia. Despite several months of treatment with erythropoietin (EPO) and Neupogen (G-CSF analogue), reticulocyte counts remained negligible (0.04–0.05 × 10⁶/µL). Ultrasonography in November revealed multiple internal organ masses, and the cat died in December 2023. Bone marrow biopsy and histopathological examination of the detected masses were not performed, as the owner declined both antemortem and postmortem sampling procedures.

**Table 5:** One-Year clinical and haematological follow-up of a cat coinfected with CMhm and FeLV (January–December 2023)

Date	Haemogram						Leukogram					PLT10³/µL	Pathogen detection		Others
	HCT%	Hgbg/dL	RBCs10⁶/µL	MCVfL	MCHCg/dL	Retic10⁶/µL	TLC10³/µL	N10³/µL	L10³/µL	M10³/µL	E10³/µL				
													CMhm	FeLV	
Jan	**16.30**↓	**5.20**↓	**2.00**↓	81.50	31.90	0.04	9.20	3.22	4.08	0.80	1.10	**170**↓	+ve	+ve	–
April	**18.00**↓	**5.60**↓	**2.85**↓	63.16	31.11	0.05	10.80	3.89	6.70	0.00	0.20	175	+ve	+ve	–
Jun	**14.70**↓	**4.70**↓	**2.10**↓	70.00	31.90	0.05	17.30	5.00	12.2	0.10	0.00	**115**↓	–	–	–
Aug	**14.30**↓	**4.40**↓	**1.97**↓	72.40	30.70	0.05	14.76	4.52	9.44	0.47	0.32	**32**↓	+ve	+ve	–
Nov	–	–	–	–	–	–	–	–	–	–	–	–	–	–	Multiple masses in internal organs by ultrasonography

*Abbreviations*: *CMhmCandidatus* Mycoplasma haemominutum, *E* eosinophils, *FeLV* feline leukaemia virus, *HCT* haematocrit, *Hgb* haemoglobin, *L* lymphocytes, *M* monocytes, *MCHC* mean corpuscular haemoglobin concentration, *MCV* mean corpuscular volume, *N* neutrophils, *PCV* packed cell volume, *PLT* platelets, *RBCs* red blood corpuscles, *Retic* reticulocytes, *TLC* total leucocytic count

Reference ranges: E, 0–0.8 × 10³/µL; Hgb, 9.8–15.4 g/dL; L, 1.5–7 × 10³/µL; M, 0–0.9 × 10³/µL; MCHC, 30–36 g/dL; MCV, 39–55 fL; N, 2.5–12.5 × 10³/µL; PCV, 30–45%; PLT, 175–600 × 10³/µL; RBCs, 5–10 × 10⁶/µL; Retic, <0.06 × 10⁶/µL; TLC, 5–19.5 × 10³/µL

↓ indicates values below the reference range; (–) indicates not tested

### Molecular characterisation and phylogenetic analysis

CMhm 16 S rRNA gene sequences generated in this study (*n* = 10), together with reference sequences retrieved from GenBank (*n* = 31), clustered into 14 nucleotide sequence types (nSTs). The largest was nST02, comprising 16 sequences. Egyptian sequences obtained in this study were distributed across five nSTs: two (nST02 and nST11) that also contained one of the two previously reported Egyptian sequences (OP023145 and OP023148), two (nST07 and nST10) that grouped with other international reference sequences, and one (nST05) that was unique to this study. Table 6 summarises the nucleotide polymorphisms defining each nST and provides the number of sequences and their corresponding GenBank accession numbers for each nST. Pairwise identity among nSTs was high (98.9–99.8%) (Table 7), and Egyptian sequences generated in this study showed 99.4–100% identity among themselves and 98.9–100% identity with reference sequences.

**Table 6:** nSTs of CMhm based on partial *16 S rRNA gene* polymorphisms

nST	Nucleotide positionᵃ													
	421	430	435	452	533	553	566	579	619	676	694	801	812	830
nST01	G	C	T	T	T	C	A	T	T	G	G	A	C	G
nST02	.	.	.	.	.	.	.	.	.	.	A	.	.	.
nST03	.	.	.	.	.	T	.	.	.	.	A	.	.	.
nST04	.	.	.	.	.	.	.	.	.	.	A	G	.	.
nST05	T	.	.	.	.	.	.	.	.	.	A	.	.	.
nST06	.	.	.	.	A	.	.	.	.	.	A	.	.	.
nST07	.	.	.	.	.	.	.	.	.	.	A	.	T	.
nST08	.	.	.	C	.	.	.	.	.	.	A	.	T	.
nST09	.	T	.	.	.	.	.	G	.	.	A	.	T	.
nST10	T	.	G	.	.	.	.	.	.	.	A	.	T	.
nST11	.	.	G	.	.	.	.	.	.	.	A	.	T	.
nST12	.	.	G	.	.	.	G	.	.	C	A	.	T	T
nST13	.	.	G	.	.	.	G	G	C	.	A	.	T	.
nST14	.	.	G	.	.	.	G	G	.	.	A	.	T	.

*Abbreviations*: *CMhmCandidatus* Mycoplasma haemominutum, *nSTs* nucleotide sequence types

^a^ Nucleotide positions are numbered relative to the CMhm California strain (GenBank accession U88564) within nST01; dots (.) indicate identity with this reference sequence

nST01 includes 5 sequences (U88564, AY150978, AY150980, KM275253, AM691834); nST02 includes 16 sequences (AY150979, AF271154, DQ157143, KM275254, EU128752, PQ604639, ON180682, OR225696, PX307892, PX307894, PX307896–PX307897, PX307900, PX30790, OP023145, EU839983); nST03–06 each contain one sequence (EU170604, KF743739, PX307898, OR225697, respectively); nST07 includes 3 sequences (DQ825452, EU839984, PX307895); nST08–09 each contain one sequence (KU852586 and DQ825446, respectively); nST10 includes 2 sequences (DQ825456 and PX307893); nST11 includes 3 sequences (PX307899, KR905448, OP023148); nST12–13 each contain one sequence (KU852587 and PQ471489, respectively); nST14 includes 4 sequences (DQ825439, KM275257, EU839985, AY529634)

Egyptian sequences generated in this study are represented within nST02, nST05, nST07, nST10, and nST11

**Table 7:** Pairwise nucleotide identity matrix among CMhm nSTs based on partial *16 S rRNA* gene sequences

	nST01	nST02	nST03	nST04	nST05	nST06	nST07	nST08	nST09	nST10	nST11	nST12	nST13	nST14
nST01	ID													
nST02	99.8	ID												
nST03	99.6	99.8	ID											
nST04	99.6	99.8	99.6	ID										
nST05	99.6	99.8	99.6	99.6	ID									
nST06	99.6	99.8	99.6	99.6	99.6	ID								
nST07	99.6	99.8	99.6	99.6	99.6	99.6	ID							
nST08	99.4	99.6	99.4	99.4	99.4	99.4	99.8	ID						
nST09	99.2	99.4	99.2	99.2	99.2	99.2	99.6	99.4	ID					
nST10	99.2	99.4	99.2	99.2	99.6	99.2	99.6	99.4	99.2	ID				
nST11	99.4	99.6	99.4	99.4	99.4	99.4	99.8	99.6	99.4	99.8	ID			
nST12	98.9	99.0	98.9	98.9	98.9	98.9	99.2	99.0	98.9	99.2	99.4	ID		
nST13	98.9	99.0	98.9	98.9	98.9	98.9	99.2	99.0	99.2	99.2	99.4	99.2	ID	
nST14	99.0	99.2	99.0	99.0	99.0	99.0	99.4	99.2	99.4	99.4	99.6	99.4	99.8	ID

*Abbreviations*: *CMhmCandidatus* Mycoplasma haemominutum, *nSTs* nucleotide sequence types

All 41 CMhm sequences clustered together as a monophyletic clade with 100% bootstrap support, distinct from Mhf and CMt sequences. However, within the CMhm clade, including the Egyptian sequences, clustering was weakly supported by low bootstrap values, and no patterns were apparent based on host species, date, or geography (Fig. 1).

## Discussion

The finding that all anaemic cats in our study had at least one concurrent disease supports the prevailing view that CMhm is mainly a secondary contributor to feline anaemia, being largely dependent on other stressors or anaemia-inducing conditions [2, 45]. This contrasts with previous reports of anaemic cats without detectable concurrent conditions [23, 27, 28], which were interpreted as evidence for a primary role of CMhm; such discrepancies may reflect undetected comorbidities or, alternatively, the presence of highly virulent strains or exposure to high infectious doses capable of causing primary disease.

Notably, many non-anaemic cats in our study also harboured concurrent diseases, indicating that the stage or severity of the condition, rather than its mere presence, is critical [3]; for example, a terminal-stage FIV infection with overt immunodeficiency may have a greater impact than subclinical infection. The significantly lower median lymphocyte counts in anaemic versus non-anaemic cats further support this hypothesis. Although total leucocyte, neutrophil, and monocyte counts were lower in anaemic cats without reaching statistical significance, the consistent downward trend suggests a generally diminished immune status in this group. However, it should also be noted that lymphopenia may additionally reflect a physiological stress response (stress leukogram) mediated by endogenous corticosteroids, rather than immunosuppression alone [46].

Flea infestation was the only concurrent condition significantly associated with CMhm-induced anaemia in the final multivariable model (*P* = 0.04, OR = 7.77), suggesting trigger–vector roles. Fleas may trigger the disease by modulating the host immune response and contribute directly to anaemia through chronic blood loss, resulting in iron deficiency, as reflected in several cats by one or more of the following laboratory features: microcytosis, hypochromia, non-regenerative reticulocyte responses, low serum iron and ferritin, elevated TIBC, and low TSAT (%).

While the vector role of fleas remains uncertain, this finding provides potential evidence supporting flea-borne transmission. Since anaemia in CMhm-infected cats typically develops during the acute phase of infection (~ 3 weeks post-exposure) [20, 22], the concurrent presence of fleas on anaemic cats provides strong evidence of recent flea-mediated exposure. In contrast, non-anaemic cats may represent chronic carriers previously exposed to fleas, in whom active exposure is no longer required. While a recent study found a statistical association between FHM species and fleas [47], most previous research failed to detect associations with current or previous exposure to fleas [6, 13, 16, 48, 49] or regular ectoparasite prophylaxis [6, 13, 19]. This discrepancy could be explained by study design: previous research compared infected versus non-infected cats, but the infected groups likely included both acutely and chronically infected animals, diluting the signal of flea association. The same principle may also explain why some studies detected associations between FHM infection and geographic or seasonal factors linked to flea prevalence [3–7, 50], while others did not [17, 48, 51, 52].

Importantly, a recent meta-analysis [53] challenged the high prevalence of FHM DNA in fleas reported in six studies [8–13], key evidence supporting their vector role, arguing that the primer designed by Jensen et al. [30] used in these studies may have caused nonspecific amplifications. The meta-analysis re-examined 67 stored individual flea samples, originally reported as 34% positive in pooled samples without sequencing in one of the six studies [13], and found FHM confirmed in only 2/67 (3%) and nonspecific bacteria in 18/67 (26.9%), concluding that prevalence is much lower than previously reported. However, three of the six original studies had sequencing-confirmed high FHM prevalence in fleas [8, 9, 12], which the meta-analysis overlooked. Its conclusions were based on decade-old samples potentially affected by DNA degradation or contamination, compared individual versus pooled samples, and did not account for the distribution of nonspecific bacteria and FHM across previously reported positive pools, rendering the revised low prevalence estimate suggested by the meta-analysis’s authors potentially unreliable [54].

The only experimental study addressing the vector role of fleas reported apparent failure of CMhm transmission; however, the study had several limitations, including a small sample size, potential variability in the immune status of cats, use of laboratory-adapted CMhm strains and fleas, and rearing fleas under suboptimal temperature and humidity conditions [55].

Notably, in this study, 8/13 anaemic cats had no fleas, suggesting that while fleas may play a significant role in transmission, they are not the sole route. Transmission in these cats could have occurred via fighting, allowing infected blood to be inoculated subcutaneously through bites, supported by the higher prevalence of FHM infection reported previously in intact males, particularly those with outdoor access [2].

The consistent association between male sex and FHM infection reported in most studies [2–4, 6, 14–18, 26, 52], contrasted with the frequent failure to detect a similar association with flea infestation [6, 13, 16, 48, 49], should not be interpreted as evidence dismissing the role of fleas. Rather, it may reflect that male sex is an inherent, fixed host characteristic that persists throughout the cat’s life, and therefore remains associated with infection regardless of its stage (acute or chronic), whereas flea exposure is transient and may be more readily detected in acutely infected cats. As Tanahara et al. [15] proposed, linking risk factors specifically to cats exhibiting clinical signs of FHM infection (anaemia) may provide a clearer and more realistic understanding of these associations.

Although other concurrent diseases did not reach statistical significance, they may still be biologically relevant, particularly those occurring at higher rates in anaemic cats (FeLV, Mhf, CMt, FIV, and hyperglycaemia). Notably, only one cat in this study was co-infected with FeLV. Given the very low overall prevalence of FeLV in our ongoing survey (10/290, 3.4%), the fact that this single FeLV-positive cat fell into the anaemic group is itself biologically significant, suggesting a potential contributory role of FeLV. Importantly, this cat presented with macrocytic, normochromic, non-regenerative anaemia and thrombocytopenia (bicytopenia) that rapidly progressed to a severe form despite long-term EPO therapy, ultimately resulting in terminal death over a few months (Table 5), raising suspicion for myelodysplastic syndrome. This observation aligns with a previous experimental study reporting that concurrent CMhm and FeLV infection can induce myelodysplastic syndrome in some cats [22]. The regenerative response and accompanying erythroid hyperplasia during recovery from CMhm-induced haemolytic anaemia could predispose FeLV-infected erythroid precursors to neoplastic transformation [22].

Beyond identifying potential triggers, the present study offers detailed insights into the characteristics of CMhm-associated anaemia at both population and individual levels and proposes mechanistic explanations for its patterns. At the population level, CMhm-associated anaemia was generally mild (mean PCV 22%, median Hgb 8.2 g/dL), normocytic–normochromic (median MCV 39.2 fL, MCHC 31.7 g/dL), and non-regenerative (median reticulocytes 0.045 × 10⁶/µL). Median RBC count (5.25 × 10⁶/µL) remained within the reference interval; despite reduced HCT and Hgb, indicating that RBC count alone can underestimate anaemia. The disease primarily involved the erythroid lineage, without any other cytopenias. A haemolytic component is supported by a mildly increased TBIL (median 0.58 mg/dL) with unchanged ALT (median 18.4 U/L), consistent with preserved hepatic bilirubin clearance and the rarity of overt icterus in practice [2].

However, the combination of a non-regenerative pattern, RBC counts and indices within the reference intervals, and inconsistent iron profiles (serum iron and ferritin concentrations within reference intervals, elevated TIBC, and low TSAT%) indicates that additional pathophysiological mechanisms beyond haemolysis are at play, especially given that all anaemic cats had concurrent diseases.

One likely mechanism is true iron deficiency, caused by chronic blood loss from fleas, stomatitis, and possibly intestinal parasitism (as reflected by elevated eosinophil counts in the anaemic group), as evidenced by elevated TIBC and low TSAT% [56–58]. Another is functional iron deficiency, likely driven by FIV and upper respiratory infections, evidenced by ferritin concentrations remaining within reference intervals despite true iron depletion [56]. The finding that serum iron concentrations were within the reference interval, despite both true and functional iron deficiencies, could be explained by haemolysis, particularly intravascular haemolysis in some cats, evidenced by hyperchromia [56, 57]. These iron deficiencies suppress the regenerative response despite ongoing haemolysis and ultimately generate a large population of microcytes, which offset the effect of larger RBCs seen in some cats with regenerative responses, thereby shifting RBC indices toward the lower end of the reference interval. This microcytosis also explains the finding of an RBC count within the reference interval despite reduced haematocrit, i.e., preserved cell numbers but smaller cell size, resulting in lower total erythrocyte volume. It should be noted that the interpretation of these iron-related findings should be made cautiously, as iron studies were performed only in the anaemic group. Therefore, further investigations, including iron profile evaluation in both anaemic and non-anaemic CMhm-infected cats, are needed to allow direct statistical comparisons between groups and better clarify the role of iron dysregulation in CMhm-associated anaemia.

To further determine whether these discrepancies reflect inter- or intra-individual variation, we examined the detailed clinicopathological profiles of each anaemic cat (Table 4), which revealed that opposing mechanisms often coexisted within the same individual, with additional discrepancies and mechanistic insights emerging. In some cats with triple haemoplasma infection, findings including haemolytic anaemia, reticulocytosis, macrocytosis, and increased TBIL supported active haemolysis with regenerative responses. In these cats, elevated ferritin concentrations likely reflected mild concurrent inflammatory conditions, such as upper respiratory infections, without apparent suppression of marrow regeneration. Markedly increased TIBC in some regenerative cases may instead reflect increased iron demand by actively regenerating marrow rather than true iron deficiency, a phenomenon also reported in human congenital haemolytic disorders accompanied by elevated serum transferrin concentrations [59].

In contrast, other cats exhibited evidence of concurrent haemolysis and iron deficiency, including reticulocytosis with microscopic microcytosis despite normocytic RBC indices. In such cases, strong regenerative responses may have been counterbalanced by preexisting microcytes, masking expected macrocytic changes. Similarly, iron profiles that appeared within reference intervals may have represented the net effect of multiple opposing processes occurring simultaneously, including true iron deficiency associated with flea infestation, haemolysis related to triple haemoplasma infection, and functional iron deficiency associated with concurrent inflammatory or viral diseases such as FIV [56–58].

Interestingly, three anaemic cats in our study exhibited microcytic hyperchromic anaemia (one regenerative, two non-regenerative), a rare pattern previously reported in a large retrospective study of 1,098 anaemic cats, in which only 21 cases (2.9%) were identified [60]. This finding is most likely attributable to analytical or pre-analytical artefacts, including in vitro haemolysis, lipaemia, icterus, Heinz body interference, erythrocyte shrinkage due to under-filled tubes, or other sample handling errors that may falsely increase MCHC values. Alternatively, in vivo haemolysis remains a possible explanation [57, 58, 61]; although CMhm infection is typically associated with extravascular haemolysis, intravascular haemolysis has been rarely reported, including two cases in a Swiss study [3].

Genetic characterisation of ten CMhm sequences in this study identified five nSTs: two corresponded to the only previously reported Egyptian sequences [29], while the remaining three were detected in Egypt for the first time, including one unique sequence among all reference strains analysed. These findings provide novel insights into the microdiversity of CMhm in Egypt and contribute to the global dataset. The absence of clustering among CMhm strains, despite including reference sequences from a wide range of hosts, times, and countries, reflects the conserved nature of the 16 S rRNA gene and confirms its limited suitability for tracing bacterial evolution or identifying sites of introduction.

There are several limitations to this study. First, the small sample size may have reduced the statistical power to detect differences between anaemic and non-anaemic cats. However, they represent all CMhm in our previous survey, allowing us to contextualise the current findings within a larger, well-characterised population. Second, as this was a cross-sectional study, the observed associations, particularly the relationship between flea infestation and CMhm-associated anaemia, cannot establish causality or temporal sequence. Third, the use of conventional PCR prevented quantification of bacterial blood load in both groups, which limits our ability to examine whether pathogen load influences disease severity or anaemia development. However, a previous study found no correlation between bacterial load and the occurrence or severity of anaemia [3]. Fourth, the absence of reticulocyte counts and iron profiles in the non-anaemic group precluded statistical comparison of these parameters with those in the anaemic group. Moreover, direct biomarkers of haemolysis, inflammation, and iron regulation were not comprehensively evaluated; therefore, the proposed mechanisms involving haemolysis, true iron deficiency, and functional iron deficiency should be considered plausible interpretations of the available clinicopathological findings rather than definitive mechanistic conclusions. Finally, targeting a conserved gene such as 16 S rRNA limited our ability to investigate pathogen-specific determinants of disease, such as virulence factors. Other genes that could provide this information are rarely reported, which limits our ability to include them in the study.

## Conclusions

The findings in this study suggest that CMhm-associated anaemia in naturally infected cats is likely a secondary condition, with its manifestation shaped by environmental triggers, host immune status, and concurrent diseases. Flea infestation emerged as the main driver of anaemia, highlighting a potential vector-mediated mechanism, while other comorbidities, such as retroviral infections and co-infections with other *Mycoplasma* species, may exacerbate or modulate disease severity. The observed anaemia was often deceptively normocytic–normochromic and non-regenerative, reflecting the complex interplay among haemolysis, true iron deficiency, and functional iron restriction induced by inflammation. Molecular characterisation revealed both previously reported and novel CMhm nSTs in Egypt, demonstrating local microdiversity without clear clustering, consistent with the conserved nature of the 16 S rRNA gene. Overall, these findings elucidate the multifactorial pathophysiology of CMhm-associated anaemia, providing mechanistic insights to enhance clinical interpretation, guide management strategies, and inform control measures in feline populations.

## Data Availability

DNA sequences generated in this study were submitted to GenBank (NCBI) following accession numbers: PX307892–PX307901.
